# Efficacy of Fertility-Sparing Treatments for Early-Stage Endometrial Cancer—Oncologic and Reproductive Outcomes: Protocol of a Systematic Review and Meta-Analysis

**DOI:** 10.3390/life15071133

**Published:** 2025-07-18

**Authors:** Márton Keszthelyi, Pál Sebok, Balázs Vida, Verita Szabó, Noémi Kalas, Szabolcs Várbíró, Lotti Lőczi, Nándor Ács, Petra Merkely, Richárd Tóth, Balázs Lintner

**Affiliations:** 1 Department of Obstetrics and Gynecology, Semmelweis University, 1082 Budapest, Hungary; psebok6@gmail.com (P.S.); vida.balazs.lajos@semmelweis.hu (B.V.); szabo.verita@gmail.com (V.S.); kalasnoemi@gmail.com (N.K.); keszthelyi.lotti.lucia@semmelweis.hu (L.L.); acs.nandor@semmelweis.hu (N.Á.); merkely.petra@gmail.com (P.M.); toth.richard@semmelweis.hu (R.T.); lintner.balazs.zoltan@semmelweis.hu (B.L.); 2 Workgroup of Research Management, Doctoral School, Semmelweis University, 1085 Budapest, Hungary; varbiroszabolcs@gmail.com

**Keywords:** endometrial cancer, fertility preservation, progesterone, hysteroscopic resection

## Abstract

Background: Endometrial cancer (EC) is the most common gynecological malignancy, increasingly affecting premenopausal women. While hysterectomy is the standard treatment, it eliminates reproductive potential, highlighting the need for effective fertility-sparing alternatives. Current ESHRE/ESGO/ESGE guidelines recommend progestin-based therapies, often with hysteroscopic resection. However, these are based on limited pharmacological options and moderate to low-quality evidence. Novel and combination therapies have shown promise but remain absent from current clinical guidelines. This review aims to evaluate the efficacy and safety of fertility-preserving treatments for early-stage EC, emphasizing the need to update current strategies based on emerging data. Methods: A systematic review and meta-analysis will follow PRISMA guidelines and the Cochrane Handbook. Eligible studies, including randomized and non-randomized designs, will assess fertility-preserving treatments for early-stage EC. Data will be extracted on complete response, recurrence, and long-term fertility outcomes. The GRADE system will assess evidence certainty. Risk of bias will be evaluated using RoB 2 for RCTs and ROBINS-I for non-randomized studies. Meta-analysis will be performed if sufficient data are available. Conclusions: This review will provide a comprehensive analysis of fertility-sparing treatments for early-stage EC, support personalized strategies, identify evidence gaps, and guide future research. Trial registration—Prospero: CRD420251032161.

## 1. Introduction

Endometrial cancer (EC) is the most common gynecologic malignancy in developed countries, with incidence rates rising among premenopausal women, particularly those under 50 years of age [1]. Approximately 7% of EC cases are diagnosed in women under 45 years of age, particularly due to rising obesity rates, polycystic ovary syndrome, and delayed childbearing [2,3]. For these women, the standard treatment—hysterectomy with bilateral salpingo-oophorectomy—presents a significant burden, as it permanently eliminates the possibility of future pregnancy [4].

Fertility-sparing approaches have evolved significantly, prioritizing uterine preservation while maintaining oncologic safety. Current ESGO/ESHRE/ESGE guidelines recommend conservative treatment, typically involving oral or intrauterine progestins, often preceded by hysteroscopic tumor resection [5]. With this approach, a complete response is achieved in up to 80% of cases within 6–12 months, yet recurrence remains common, occurring in nearly one-third of patients. While some combination therapies (e.g., metformin or GnRH analogs) have demonstrated improved outcomes, recommendations remain based on monotherapy or limited pharmacologic options, and only a minority is supported by high-level evidence [6]. Despite these advances, challenges persist in optimizing treatment duration, preventing recurrence, and managing higher-risk subgroups, such as those with genetic predispositions or grade 2 histology [7].

From a mechanistic standpoint, progestin therapies—whether administered orally or via a levonorgestrel-releasing intrauterine device (LNG-IUD)—act through progesterone receptor binding in endometrial glandular and stromal cells, leading to antiproliferative effects, glandular atrophy, stromal decidualization, and induction of apoptosis. Both routes of administration share similar biological mechanisms, though LNG-IUDs provide higher local concentrations with fewer systemic side effects. Progestin therapy downregulates estrogen receptor signaling and modulates pathways such as p21 and Bcl-2, contributing to tumor regression [8]. Combination strategies may further enhance efficacy; metformin, commonly used in insulin-resistant patients, inhibits the PI3K/AKT/mTOR pathway, alters cellular metabolism, and has been shown to increase progesterone receptor expression and restore sensitivity to progestin-induced apoptosis in resistant endometrial cancer cells [9]. GnRH analogs suppress ovarian estrogen production by downregulating pituitary gonadotropins, thereby inhibiting endometrial cancer cell proliferation by binding to tumor-expressed GnRH receptors, activating G-protein αi–mediated pathways that suppress EGF and estrogen signaling, leading to cell cycle arrest. They may also modulate apoptosis and are used clinically for estrogen suppression in fertility-sparing treatments or as targeted therapy in GnRH receptor-positive tumors [10]. These synergistic mechanisms offer a biologically plausible rationale for multimodal fertility-sparing approaches.

This review will evaluate the efficacy, safety, and reproductive outcomes of current fertility-sparing strategies, with a focus on hormonal, surgical, and combined modalities. By synthesizing evidence on response rates, relapse risks, and live birth outcomes, we aim to clarify optimal patient selection criteria and therapeutic protocols for this growing patient population.

Although progress has been made recently, head-to-head comparisons between monotherapies and combination treatments are limited. The effectiveness of these approaches—especially in terms of complete response rates, recurrence patterns, and long-term reproductive outcomes—requires more comprehensive evaluation. As such, we recognize the need for an updated meta-analysis that includes all available treatment combinations, which may be useful for future clinical practice and support revisions of existing guidelines.

To ensure methodological accuracy and minimize bias, we will structure this study according to the PICO framework.

The population (P) comprises premenopausal women diagnosed with stage 1A endometrial cancer who are undergoing fertility-preserving treatment.

The interventions (I) are LNG-IUD, oral progestins, oral progestins + LNG-IUD, oral progestins + hysteroscopic resection, LNG-IUD + GnRH analogs, LNG-IUD + hysteroscopic resection, and oral progestins + metformin.

The comparator (C) group will include the same treatment modalities to allow comparative analysis: LNG-IUD, oral progestins, oral progestins + LNG-IUD, oral progestins + hysteroscopic resection, LNG-IUD + GnRH analogs, LNG-IUD + hysteroscopic resection, and oral progestins + metformin.

The outcomes (O) of interest will include CR rates, recurrence rates, and long-term fertility outcomes.

## 2. Review Question

What is the most effective fertility-preserving treatment strategy that provides an optimal balance between oncologic safety and reproductive outcomes—defined as the highest rates of complete response, pregnancy, and live birth, alongside the lowest recurrence rate—in women diagnosed with early-stage endometrial cancer?

## 3. Materials and Methods

This protocol was developed in alignment with the Preferred Reporting Items for Systematic Reviews and Meta-Analyses Protocols (PRISMA-P) checklist [11]. The methodological framework outlined in the Cochrane Handbook for Systematic Reviews of Interventions standards was followed. The protocol was submitted to PROSPERO on 13 April 2025.

### 3.1. Eligibility Criteria

This review will include both randomized controlled trials and non-randomized studies, such as prospective and retrospective cohort designs, published in English. Studies must evaluate fertility-preserving treatment strategies in patients diagnosed with early-stage endometrial cancer. The literature search encompassed publications from the inception of each database through 30 April 2025.

### 3.2. Information Sources

A systematic and comprehensive literature search will be conducted across multiple databases, including the Cochrane Central Register of Controlled Trials (CENTRAL), EMBASE, Scopus, Web of Science, and PubMed. There are no restrictions on the date of or the status of publications. In addition, the reference lists of all included studies will be manually reviewed to identify any further eligible publications.

### 3.3. Search Strategy

A thorough literature search will be performed using a combination of Medical Subject Headings (MeSH) terms and free-text keywords to identify all pertinent studies. The search strategy included the following terms: (“endometrium” OR “endometrial” OR “endometrioid” OR “endometr*”) AND (“hyperplasia” OR “hyperplas*” OR “thick*”) AND (“gestagen” OR “gest*” OR “progesterone” OR “progest*” OR “medroxyprogesterone acetate” OR “medroxyprogesterone” OR “progesterone derivative” OR “megestrol acetate” OR “dienogest derivative” OR “levonorgestrel” OR “hydroxyprogesterone” OR “medrogestone” OR “megestrol” OR “desogestrel derivative” OR “drospirenone” OR “dydrogesterone” OR “intrauterine device” OR “IUD” OR “metformin” OR “GnRHa” OR “gonadotropin-releasing hormone agonist” OR “GnRH analogue” OR “hysteroscopy” OR “hysteros*” OR “hysteroscopic resect*”).

### 3.4. Study Records

#### 3.4.1. Data Management

All retrieved search results will be imported into EndNote (version X9.3.3; Clarivate Analytics, Ann Arbor, MI, USA), a reference management software designed to facilitate the organization and management of citations. EndNote will be used to efficiently manage references and identify any duplicate records, which will then be removed to ensure data integrity. Following de-duplication, the remaining studies will undergo a structured screening and selection process using Rayyan (version 1.5.3; Rayyan Systems Inc., Cambridge, MA, USA), a web-based application specifically developed to assist in conducting systematic reviews. Rayyan enables multiple reviewers to perform independent and blinded screening, thereby enhancing the objectivity and reliability of the selection process.

#### 3.4.2. Selection Process

**Figure 1:**
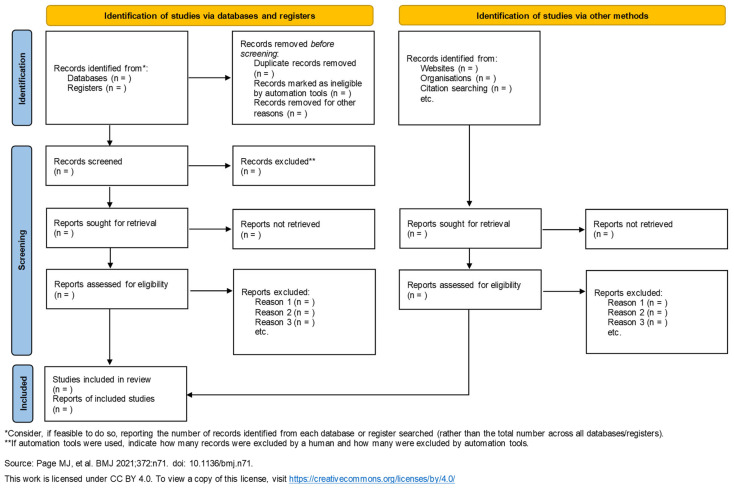
The PRISMA flow diagram summarizing the key methodological steps [11].

The initial phase of study selection will involve an independent screening of titles and abstracts by two reviewers to identify potentially relevant studies. This will be followed by a full-text review of the shortlisted articles to assess their eligibility based on predefined inclusion and exclusion criteria. Any discrepancies or disagreements that arise between the two reviewers during this process will be addressed through detailed discussion and consensus. In instances where a resolution cannot be achieved, a third reviewer will be consulted to provide an impartial assessment and make the final decision. Furthermore, a thorough and transparent record will be maintained for all full-text articles excluded at this stage, clearly outlining the specific rationale for their exclusion to ensure accountability and reproducibility of the review process (Figure 1).

#### 3.4.3. Data Collection Process

Data will be independently extracted by two reviewers into standardized Excel spreadsheets (Microsoft Excel, Office 365; Microsoft Corporation, Redmond, WA, USA). Any discrepancies or inconsistencies in the extracted data will be addressed through discussion between the reviewers or, if necessary, through consultation with a third reviewer to reach a resolution.

### 3.5. Data Items

Data extraction will be performed independently by two reviewers using a pre-designed, standardized data collection form to ensure consistency and accuracy across all included studies. Key study-level information will be recorded, including the name of the first author, year of publication, geographic location of the study, study design (e.g., randomized controlled trial, cohort study), and total sample size. Specific details on the number of participants diagnosed with endometrial cancer and the number included in the final analysis will also be captured.

Comprehensive information on interventions will be extracted, including the type of fertility-preserving treatment administered and, where applicable, the comparator or control intervention. Additional variables will include duration of follow-up and baseline characteristics of participants, such as mean or median age and body mass index (BMI).

Key clinical outcomes to be extracted will include complete response rates, recurrence rates, progression to endometrial cancer (if applicable), time to clinical response, pregnancy rates, live birth rates, and method of conception, categorized as either natural or via assisted reproductive technologies (ART). In studies that report subjective measures such as pain or quality-of-life outcomes using varying scales, appropriate data normalization or transformation will be undertaken to facilitate meaningful comparisons across studies.

If essential outcome data are missing, incomplete, or unclear, efforts will be made to contact the corresponding authors of the original studies to request clarification or additional information, thereby ensuring the robustness and completeness of the data set.

### 3.6. Outcomes and Prioritization

The main focus of this review is to evaluate the rate of complete response (CR), which is characterized by the absence of disease confirmed through histological analysis—specifically, no signs of atypical hyperplasia or carcinoma present in endometrial tissue after fertility-preserving therapy. Additional outcomes under consideration include the frequency of disease recurrence after an initial CR, the rate at which the condition progresses to more severe stages, and important reproductive indicators such as the likelihood of becoming pregnant post-CR, the live birth rate, and whether conception occurred naturally or through assisted reproductive techniques.

Where sufficient data are reported (i.e., where at least three independent studies provide extractable and comparable data for a given subgroup variable), the review will also explore how patient-specific variables—such as age, body mass index, and treatment type (e.g., oral progestins, levonorgestrel-releasing intrauterine system, or a combined regimen)—may influence outcomes. These analyses aim to provide deeper insight into the relative effectiveness and reproductive success associated with various therapeutic approaches.

### 3.7. Risk of Bias in Individual Studies

To ensure methodological rigor and minimize subjectivity in the appraisal of included studies, two reviewers will independently assess the risk of bias for each study. For non-randomized studies, the Risk Of Bias In Non-randomized Studies of Interventions (ROBINS-I) tool will be employed. ROBINS-I is designed to evaluate bias across seven critical domains: confounding variables, classification of interventions, selection of participants (whether for study inclusion or analytic comparison), deviations from intended interventions, completeness of outcome data, accuracy in outcome measurement, and the potential for selective reporting of results.

For studies utilizing random allocation, the revised Cochrane Risk of Bias Tool for Randomized Trials (RoB 2) will be applied. This tool systematically evaluates five principal domains: the randomization process itself, adherence to intended intervention protocols, completeness and integrity of outcome data, reliability of outcome measurement, and risk of selective reporting or publication bias.

Within both tools, each domain is assessed through a structured series of signaling questions designed to uncover relevant evidence. Responses to these questions guide an algorithm that categorizes each domain as having “low risk”, “some concerns”, or “high risk” for each domain. These individual domain-level assessments will then be synthesized to generate an overall risk of bias rating, using the same three-tiered scale.

In instances where discrepancies arise between the two reviewers regarding risk of bias judgments, the matter will be resolved through discussion. If consensus cannot be reached, a third, independent reviewer will be consulted to provide an objective opinion and finalize the rating. This process ensures consistency, transparency, and objectivity in the quality assessment of all included studies.

### 3.8. Data Synthesis

A meta-analysis will be conducted if the included studies demonstrate an adequate level of consistency in their characteristics and findings. Both narrative and statistical data synthesis will be applied. At least three eligible studies will be necessary to proceed with the meta-analysis. A random-effects model will be used to aggregate effect sizes, following a frequentist framework. For binary outcomes, risk ratios (RRs) will be calculated with corresponding 95% confidence intervals (CIs). Event proportions will be pooled separately within each comparison group.

In the case of continuous outcomes, either the mean difference (MD) or the median difference (MedD) will be reported, depending on data format, with 95% CI for each. If only quartile data are available, mean and standard deviation (SD) values will be estimated assuming a normal or lognormal distribution; if such assumptions cannot be met, pooling will rely on medians. Risk ratios from raw data will be pooled using the Mantel–Haenszel approach, with its exact variant employed to account for cells with zero events. If individual-level data are unavailable, the inverse variance method will be used to calculate pooled estimates of both RR and MD.

To improve the robustness of confidence intervals, the Hartung–Knapp adjustment will be used where it yields more conservative estimates. The heterogeneity variance (τ^2^) will be estimated via the restricted maximum likelihood method, and its confidence intervals will be calculated using the Q-profile technique. Study-level heterogeneity will be quantified using the I^2^ statistic developed by Higgins and Thompson. Results will be interpreted as statistically significant if the CI does not encompass the null value. Findings will be illustrated with forest plots, and prediction intervals will be included where relevant. Model diagnostics and outlier detection will be carried out using a combination of visualizations and statistical influence measures. All computations will be performed using R software (R Core Team, Austria), version 4.4.2.

### 3.9. Assessment of Meta-Biases

To evaluate the presence of meta-biases, we will assess the potential for publication bias and selective reporting across studies. For outcomes in ten or more studies, we will visually inspect funnel plots for asymmetry, which may suggest small-study effects or publication bias. In addition, Egger’s regression test and Begg’s test will be applied to statistically assess funnel plot asymmetry, with a significance threshold of *p* < 0.10. In cases where reporting bias is suspected, we will compare outcomes reported in published articles with those listed in study protocols or trial registries, if available. Furthermore, we will examine whether selective non-reporting of outcomes may be present. Where applicable, subgroup analyses and sensitivity analyses will be used to explore the robustness of findings in light of any detected biases.

### 3.10. Confidence in Cumulative Evidence

To evaluate the overall confidence in the evidence for each main and secondary outcome, we will utilize the GRADE methodology. This framework systematically assesses evidence quality across five core domains: study limitations (risk of bias), heterogeneity (inconsistency), relevance of evidence (indirectness), precision of estimates (imprecision), and potential for publication bias. Based on these criteria, each outcome will be categorized as high, moderate, low, or very low certainty. Evidence from randomized trials will initially be considered high certainty but may be downgraded if concerns are identified in any of the assessed domains. Conversely, evidence from non-randomized studies will start at low certainty and may be upgraded in specific circumstances—such as when large effect sizes are observed, when a clear dose–response relationship exists, or when all likely confounding would serve to diminish the observed effect. Key findings and corresponding quality ratings will be summarized using GRADE-pro GDT software in the form of Summary of Findings tables. All judgments regarding changes in certainty levels will be clearly justified and fully documented [12,13,14].

## 4. Discussion

This review protocol incorporates a number of significant methodological strengths. It adheres to established standards, including the PRISMA framework. The methodology is guided by the principles outlined in the Cochrane Handbook for Systematic Reviews of Interventions, ensuring methodological transparency and reproducibility. The certainty of evidence will be appraised using the GRADE framework, further enhancing the credibility and interpretability of the findings. By systematically compiling and analyzing comparative data on fertility-preserving therapies for endometrial cancer, this review seeks to deliver a comprehensive evaluation of both oncologic and reproductive outcomes associated with conventional and novel treatment options.

Several real-world studies have reported encouraging clinical outcomes in early-stage endometrial cancer. In a 15-year prospective cohort study by Falcone et al., 28 young women underwent hysteroscopic resection followed by progestin therapy. The complete response rate was 96.3% among patients with grade 1 disease, with a recurrence rate of only 7.7%. Of those who attempted conception, 93.3% achieved pregnancy and 86.6% had a live birth [15]. Similarly, Fang et al. evaluated 47 patients with early-stage endometrial cancer or atypical hyperplasia and found that treatment with a levonorgestrel-releasing intrauterine system (LNG-IUS) combined with oral progesterone led to a significantly higher fertility rate (55% vs. 20%) and fewer adverse effects compared to LNG-IUS alone [16]. These findings support the efficacy and feasibility of multimodal fertility-sparing approaches in clinical practice. One of the main challenges in the existing body of research is the substantial variation in treatment approaches—particularly in terms of dosage schedules and the use of combination therapies. This clinical heterogeneity is further compounded by inconsistencies in follow-up duration, inclusion criteria, and reporting of outcomes across studies, which may pose significant challenges in conducting meaningful meta-analyses and limits the generalizability of results to diverse clinical settings. Although prior reviews have established progestin therapy as an effective first-line option, the lack of robust comparative data on multimodal strategies—such as incorporating hysteroscopic intervention or metabolic co-treatments—makes it difficult to define the most effective treatment algorithms with confidence.

This review aims to bridge the gap in the literature by gathering the latest evidence on both single-agent and combination treatment approaches. Preliminary findings from retrospective analyses suggest that multimodal strategies—such as hysteroscopic resection combined with oral progestins or levonorgestrel-releasing intrauterine devices, or the addition of GnRH analogs—may enhance complete response rates and reduce time to remission [15,16]. Moreover, increasing attention is being paid to the potential adjunctive role of metformin, particularly in patients with insulin resistance or metabolic syndrome, as a means of improving both oncologic and reproductive outcomes.

In summary, the results of this review are expected to provide meaningful contributions to clinical practice and the refinement of treatment guidelines for fertility preservation in endometrial cancer. By consolidating the existing evidence, this review may encourage the adoption of more individualized treatment approaches and provide guidance for the design of future prospective studies with standardized outcome measures and extended follow-up periods.

## Data Availability

The original contributions presented in this study are included in the references. Further inquiries can be directed to the corresponding author(s).

## Abbreviations

ART	Assisted Reproductive Technologies
BMI	Body Mass Index
CR	Complete Response
EC	Endometrial Cancer
GnRH	Gonadotropin-Releasing Hormone
LNG-IUD	Levonorgestrel-Releasing Intrauterine Device
MD	Mean Difference
PRISMA-P	Preferred Reporting Items for Systematic Reviews and Meta-Analyses Protocols
RCT	Randomized Controlled Trial
RR	Risk Ratio
SD	Standard Deviation
